# Genetic and epigenetic changes in primary metastatic and nonmetastatic colorectal cancer

**DOI:** 10.1038/sj.bjc.6603337

**Published:** 2006-09-12

**Authors:** E Miranda, A Destro, A Malesci, E Balladore, P Bianchi, E Baryshnikova, G Franchi, E Morenghi, L Laghi, L Gennari, M Roncalli

**Affiliations:** 1 Molecular Genetics Laboratory, Istituto Clinico Humanitas, University of Milan, Via Manzoni 56, Rozzano, Milano 20089, Italy; 2 Departement of Gastroenterology, Istituto Clinico Humanitas, University of Milan, Via Manzoni 56, Rozzano, Milano 20089, Italy; 3 Clinical Trial Office, Istituto Clinico Humanitas, University of Milan, Via Manzoni 56, Rozzano, Milano 20089, Italy; 4 Departement of Gastroenterology, Istituto Clinico Humanitas, University of Milan, Via Manzoni 56, Rozzano, Milano 20089, Italy; 5 Departement of Surgery, Istituto Clinico Humanitas, University of Milan, Via Manzoni 56, Rozzano, Milano 20089, Italy; 6 Departement of Pathology, Istituto Clinico Humanitas, University of Milan, Via Manzoni 56, Rozzano, Milano 20089, Italy

**Keywords:** colorectal cancer, genetic and epigenetic alterations, metastogenic phenotype

## Abstract

Colorectal cancer (CRC) develops as multistep process, which involves genetic and epigenetic alterations. *K-Ras*, *p53* and *B-Raf* mutations and *RASSF1A*, *E-Cadherin* and *p16INK4A* promoter methylation were investigated in 202 CRCs with and without lymph node and/or liver metastasis, to assess whether gene abnormalities are related to a metastogenic phenotype. *K-Ras*, *B-Raf* and *p53* mutations were detected in 27, 3 and 32% of the cases, with *K-Ras* mutations significantly associated with metastatic tumour (*P*=0.019). *RASSF1A*, *E-Cadherin* and *p16INK4A* methylation was documented in 20, 44 and 33% of the cases with *p16INK4A* significantly associated with metastatic tumours (*P*=0.001). Overall, out of 202 tumours, 34 (17%) did not show any molecular change, 125 (62%) had one or two and 43 (21%) three or more. Primary but yet metastatic CRCs were prevalent in the latter group (*P*=0.023) where the most frequent combination was one genetic (K-Ras in particular) and two epigenetic alterations. In conclusion, this analysis provided to detect some molecular differences between primary metastatic and nonmetastatic CRCs, with *K-Ras* and *p16INK4A* statistically altered in metastatic tumours; particular gene combinations, such as coincidental *K-Ras* mutation with two methylated genes are associated to a metastogenic phenotype.

Colorectal cancer (CRC) is one of the most common malignant neoplasm worldwide. The prognosis is significantly affected by the tumour stage that mainly rests on the ability to metastasise to lymph nodes and/or to the liver. Indeed, the 5-year-survival is over 90% for Duke's stage A, but only 5% for Duke's stage D (de la Chapelle, 2004). Sequential and additional genetic alterations affecting several proto-oncogenes and tumour suppressor genes have been recognised in the pathogenesis of CRC. Less known are the molecular events responsible for the progression and metastatisation of CRC; it is reasonable that the molecular profile of primary but yet metastatic CRC may be helpful to understand the molecular basis of extracolic tumour spread and to define therapeutic strategies against ‘metastogenic’ genes. Previous studies targeted to ascertain the molecular progression of CRC investigated genetic abnormalities of proto-oncogenes (*K-Ras*) and tumour suppressor genes (*p53* and *APC*) or, alternatively, molecular epigenetic changes (*E-Cadherin*, *p16INK4A*, *DAPK, RASSF1A* and *APC*) (Smith *et al*, 2002; Lee *et al*, 2004). The present study was aimed to investigate both genetic and epigenetic changes occurring in CRC by comparing the molecular profiles of primary metastatic and nonmetastatic tumours. We selected for study the mutational status of *K-Ras*, *B-Raf* and *p53* and the epigenetic status of *RASSF1A*, *E-Cadherin* and *p16INK4A*.

Abnormalities of *K-Ras* are key events in colorectal carcinogenesis and mutations of the gene arise early during the colonic transformation in 20–50% of the tumours (Bazan *et al*, 2002). *K-Ras* has been also implicated in the process of tumour invasion and metastasis (Giehl, 2005). Mutations that prevalently occur at codon 12, prevent efficient GTP-hydrolysis and thus render the protein in an activated state. As a result, multiple Ras effector pathways, which control fundamental biological processes such as proliferation, apoptosis and cell motility become constitutively activated and/or deregulated (Pollock *et al*, 2005). The best characterised Ras effectors are the Raf serine/threonine kinases (*A-Raf*, *B-Raf* and *Raf-1*) that upregulate the MAP kinase cascade (Kolch, 2000). Davies *et al* (2002) recently reported that *B-Raf* mutations play a pivotal role in the oncogenesis of melanoma and colorectal carcinoma. The majority of analysed samples exhibited one specific *B-Raf* alteration that caused the exchange of valine to glutamate, a negatively charged amino acid, at position 599 (V599E) in the kinase domain of the protein. Interestingly, it has been shown that this alteration causes the growth of carcinoma cell lines independent from Ras activation.

The *p53* suppressor gene encodes a nuclear phosphoprotein that regulates cell cycle and apoptosis. Mutations in this gene constitute some of the most frequently occurring genetic changes at human malignancies and are thought to be late development in the adenoma–carcinoma sequence in CRC (Fearon and Vogelstein, 1990). Mutations in this gene increase the protein half-life and are often associated with protein overexpression in the nucleus (Remvikos *et al*, 1990). Mutations most commonly occur in the highly conserved region (exons 5–8), which encodes the DNA-binding domain (Zhang *et al*, 1997).

During the last decade, epigenetic changes have been reported in many cancers and they are now recognised to be at least as common as genetic changes. Hypermethylation of selected CpG sites within CpG islands in the promoter region of tumour suppressor genes is associated with loss of gene expression and is observed in both physiological conditions and neoplasia (Kim *et al*, 2005b). By inactivating various tumour suppressor genes, this epigenetic modification can affect many important cellular processes, such as cell proliferation, apoptosis, invasion and metastasis.

RASSF1 proteins are potential Ras effectors and the major isoform, *RASSF1A*, is an important human tumour suppressor protein acting at G1/S phase of cell-cycle progression (Dammann *et al*, 2003). This protein can induce cycle arrest by cyclin D1 inhibition. The *RASSF1A* locus at 3p21.3 is methylated at high frequency in a variety of solid tumours including CRC, where this gene has been analysed as an alternative marker to downregulate Ras pathway (Pizzi *et al*, 2005).

*E-Cadherin* belongs to a family of Ca^2+^-dependent adhesion molecules that mediate intercellular contacts critical to morphogenesis and the maintenance of tissue structure (Takeichi, 1995). Reduced expression of E-Cadherin owing to aberrant CpG island hypermethylation has been regarded as one of the main molecular events involved in the dysfunction of the cell–cell adhesion system (Darwanto *et al*, 2003), in invasion and metastasis (Garinis *et al*, 2002). Recently, it has been shown that loss of E-Cadherin expression in a transgenic mouse model is associated with the development of invasive colorectal carcinoma from well-differentiated adenomas (Wheeler *et al*, 2001).

*p16INK4A* gene is one of the most frequently inactivated tumour suppressor genes in human cancer (Kamb *et al*, 1994). The *p16INK4A* product, an inhibitor of cyclin-dependent kinases 4 and 6, is capable of preventing Rb phosphorylation, thus blocking cells in the G1 phase of the cell cycle (Serrano *et al*, 1996). It is known that *p16INK4A* expression in colon cancer cells is repressed by methylation at the CpG island of promoter, but *in vivo* silencing of *p16INK4A* methylation has not been widely investigated (Kim *et al*, 2005a).

We designed a study aimed to compare genetic and epigenetic changes occurring in primary advanced nonmetastatic CRCs as opposed to primary advanced metastatic CRCs. The target was to establish in CRCs if and which genetic and epigenetic molecular abnormalities, among a number of selected genes, are associated to a metastogenic phenotype.

## MATERIALS AND METHODS

### Patients

This study includes 202 consecutive primary advanced metastatic and nonmetastatic CRCs surgically removed from 1997 to 2002. Table 1 reports the main clinico-pathological features of the series. We elected to study and to compare two homogeneous groups of CRC cases, namely those invading through muscolaris mucosa into subsierosa (or into nonperitonealised pericolic or perirectal tissues) with and without lymph nodes and/or liver metastases. In such a way, the only significant morphological and biological variable between the two groups was the presence of metastatic deposits. Accordingly, these two groups will be referred to throughout the paper as metastatic (M+) and nonmetastatic (M−) tumours.

### DNA extraction

Samples were fixed in formalin and embedded in paraffin. Paraffin blocks were cut into several 2 *μ*m sections. One section from each tissue was stained with hematoxylin–eosin to distinguish neoplastic tissue from the nonmalignant counterpart. After manual dissection, DNA was extracted from the unstained section of the malignant tissue as described previously (Roncalli *et al*, 2002). Briefly, tissue sections were deparaffinised using 100% xylene (Sigma, Saint Louis, MO, USA) followed by 100% ethanol. The pellet was then resuspended in a buffer containing proteinase K (Finnzyme, Espoo, Finland), and DNA was extracted with phenol–chloroform (Sigma, Saint Louis, MO, USA) followed by ethanol precipitation. Finally, precipitated DNA was resuspended in 100 *μ*l of water. DNA was quantified spectrophotometrically, and 200 ng were used as a template for each PCR amplification.

### *K-Ras* and *B-Raf* mutation (PCR–RFLP)

Mutations at codon 12 of the *K-Ras* gene and at codon 599 of the *B-Raf* gene were detected by PCR–RFLP that identifies codon 12 and 599 mutations but not the specifically altered nucleotide. *K-Ras* codon 12 mutations were detected using the *Bst*NI restriction enzymes (New England Biolabs Inc., Beverly, MA, USA). Briefly, 200 ng of DNA were used as template for the first PCR, which consisted of a 50 *μ*l volume containing *Taq* DNA polymerase (1 U; Finnzyme, Espoo, Finland), deoxynucleotide triphosphates (0.2 mM; Finnzyme, Espoo, Finland), reaction buffer (5 *μ*l; Finnzyme, Espoo, Finland), the primers forward (0.2 pmol *μ*l^−1^; Proligo, Boulder, CO, USA): 5′-ACTGAATATAAACTTGTGGTAGTTGGACCT-3′ and reverse (0.2 pmol *μ*l^−1^; Proligo, Boulder, CO, USA): 5′-TCAAAAGAATGGTCCTGCACCAG-3′. The forward primer creates the restriction site for *Bst*NI, which is lost when *K-Ras* is mutated at codon 12.

For amplification, a DNA thermocycler (MBS 0.2S, Hybaid, Ashford, UK) was used. Cycling conditions of the first PCR were as follows: initial denaturation (4 min at 94°C), followed by 40 cycles of denaturation (30 s at 94°C), annealing (15 s at 53°C), and elongation (30 s at 72°C). An elongation of 10 min at 72°C followed the last cycle; at the end, samples were kept at 4°C. Twenty microlitres of the PCR reaction were then digested with *Bst*NI for 4 h at 60°C in a total volume of 50 *μ*l. When codon 12 is wild-type, the PCR product contains a restriction site for *Bst*NI, and digestion yields bands of 128 and 29 bp. If there is a mutation in either of the first two bases of codon 12, the mutant PCR fragment will not be cut and will remain at its original size of 157 bp. Digested products were visualised by electrophoresis on a 3% agarose gels (containing 0.5 *μ*g ml^−1^ ethidium bromide). Positive (SW480 cell line) and negative controls (Human Placental DNA) for the mutation and controls for carryover DNA contamination were included in every experiment. *B-Raf* V599E mutations were analysed first by amplification using primers forward (0.2 pmol *μ*l^−1^; Proligo, Boulder, CO, USA): 5′-CTGTTTTCCTTTACTTACTACACCTCAGATA-3′ and reverse (0.2 pmol *μ*l^−1^; Proligo, Boulder, CO, USA): 5′-CTCAATTCTTACCATCCACAAAATG-3′. After 40 cycles of amplification (annealing temperature 55°C), amplified DNA was subjected to *Tsp*RI (New England Biolabs, Beverly, MA, USA) restriction digestion because this mutation abrogates a restriction site present in the wild-type sequence. After incubation with the enzyme at 65°C overnight, the products were analysed on 3% agarose gels. Special care was taken to directly load the samples at high temperature (70°C) and in the presence of formamide (0.2%) to avoid reannealing of the restricted products (*Tsp*RI leaves a nine-base 3′ overhang). DNA containing the wild-type (Human Placental DNA) or the mutated T1976 sequence (HT29 cell line) was always included as controls.

### *P53* mutation

We used both SSCP and immunohistochemistry.

#### SSCP

By SSCP we identified samples with altered *p53* migratory pattern, by gene mutation or polymorphism. Briefly, PCR fragments were generated from 200 ng of genomic DNA in a 50 *μ*l PCR reaction buffer. The primers (0.4 pmol *μ*l^−1^; Proligo, Boulder, CO, USA) and the condition for PCR are described in the Table 1 of Supplementary Appendix available on the *BJC* website. DNA containing the wild-type p53 (Human Placental DNA) was always included as controls. For SSCP analysis, PCR product was mixed with an equal volume of loading buffer containing 100% formamide (Sigma, Saint Louis, MO, USA), 0.05% xylene cyanol (Sigma, Saint Louis, MO, USA), and 0.05% bromphenol blue (Amersham Bioscience Corporation, Buckinghamshire, UK); heated at 96°C for 10 min and put on ice before being loaded on 37.5% TBE polyacrylamide gels and electrophoresed for 1 h at 100 V and 4–5 h at 300 V. The gels were silver stained with DNA Silver Staining Kit (Amersham Bioscience Corporation, Buckinghamshire, UK) according to the manufacturer's recommendations.

#### Immunohistochemistry

To analyse p53 expression, formalin-fixed paraffin-embedded tissue sections (2 *μ*m) were used, deparaffinised and exposed to an antigen retrieval system (1 mM EDTA, pH 8, and 98°C for 30 min) before being incubated with the specific p53 antibody (1:1000, Ab-2, Calbiochem; Oncogene Research Products, Cambridge, MA, USA). Endogenous peroxidase was blocked with 3% hydrogen peroxide for 20 min at room temperature. Primary mouse monoclonal antibody was applied for 1 h at room temperature. Reactive sites were identified with secondary antibody (HRP rabbit/mouse, ChemMate DAKO Envision, Carpinteria, CA, USA) for 30 min at room temperature. Immunoperoxidase staining, using diaminobenzidine as chromogen, was carried out (DAB+chromogen X-50, ChemMate, DAKOCytomation, Carpinteria, CA, USA). The slides were counterstained with hematoxylin (Harris Hematoxylin, DiaPath, Microstain Division, Martinengo, BG, Italy). An abnormal SSCP pattern coincidental with p53 nuclear immunoreactivity was taken as indicative of cases with *p53* mutation. Indeed, an altered SSCP pattern may be merely related to gene polymorphism and as such being not indicative of an abnormally mutated gene. On the other hand, the mere immunocytochemical detection of p53 gene product may also be due to an increased protein stabilisation uncoupled from mutation (Cripps *et al*, 1994). Therefore, the less expensive, quick and efficient way to detect p53 mutations is to associate an abnormal SSCP pattern with the nuclear immunocytochemical detection of the gene product.

### *RASSF1A*, E- Cadherin and *p16INK4A* methylation

As previously reported (Roncalli *et al*, 2002), 1 *μ*g of genomic DNA in a volume of 50 *μ*l was denatured by NaOH for 10 min at 37°C; 30 *μ*l of 10 mM hydroquinone (Sigma, Saint Louis, MO, USA) and 520 *μ*l of 3 M sodium bisulphite (Sigma, Saint Louis, MO, USA) at pH 5, both freshly prepared, were added and mixed, and samples were incubated at 50°C for 16 h. Modified DNA was purified using the Wizard DNA purification resin according to the manufacturer's instruction (Promega, Milano, Italy) and eluted into 50 *μ*l of water. Modification was completed by NaOH treatment (final concentration 0.3 M) for 5 min at room temperature, followed by ethanol precipitation. DNA was resuspended in water and used immediately or stored at −20°C. Polymerase chain reaction was performed separately with methylation-specific primers (0.12 pmol *μ*l^−1^; Proligo, Boulder, CO, USA) and unmethylation primers (0.12 pmol *μ*l^−1^; Proligo, Boulder, CO, USA) for each gene (Supplementary Appendix, Table 1). Unmethylated (Human Placental DNA) and methylated DNA (LoVo, COLO320 and HepG2 cell lines for *RASSF1A, E-Cadherin* and *p16INK4A*, respectively) were used as MSP controls. Polymerase chain reaction products were analysed on 2% agarose gels, stained with ethidium bromide (0.5 *μ*g ml^−1^), and visualised under UV illumination. A sample was classified as methylated whenever a band, corresponding to the molecular weight of the methylated PCR product, had a thickness and staining intensity equal or greater than that of the unmethylated PCR product. Figure 1 of Supplementary Appendix available on the *BJC* website illustrates an example of methylated and unmethylated PCR products.

### Statistical analyses

Data were described as number and percentage or means and s.d., whether appropriate. Differences in frequencies of variables were tested using the *χ*^2^ test (Pearson or Fisher, if appropriate), and differences in mean levels of variables were tested using the *t*-test. A *P*-value of 0.05 was considered statistically significant. All variables found to have a *P*-value ⩽0.1 in the univariate evaluation were considered to be candidates for a subsequent stepwise multivariate logistic regression analysis. Data analysis was performed using STATA9.

## RESULTS

### Genetic changes

The mutational status of *K-Ras*, *B-Raf* and *p53* in 202 cases of CRC is shown in Table 2a.

#### *K-Ras*

Codon 12 *K-Ras* mutations were found in 54 cases (27%). By univariate analysis, *K-Ras* mutations were significantly associated with M+ tumours (*P*=0.016) and more frequent detectable in the right side (*P*=0.088). Multivariate analysis confirmed the association between *K-Ras* mutations and M+ tumours (OR 2.37, 95% CI, 1.15–4.88, *P*=0.019).

#### *B-Raf*

Seven cases (3%) showed *B-Raf* mutation but none of these had a coincidental mutation in the *K-Ras* gene nor a statistical association with the other clinico-pathological variables.

#### *p53*

An altered SSCP pattern was identified in 91 (45%) out of 202 samples, whereas p53 nuclear immunoreactivity was detected in 115 (57%) out of 202 cases. A coincidental abnormal SSCP migratory pattern and p53 nuclear immunoreactivity was documented in 65 (32%) out of 202 cases.

No association was found in cases showing both genetic and phenotypic *p53* abnormalities with the clinico-pathological parameters under study.

### Epigenetic changes

All cases showing PCR products for methylated DNA sequences also exhibited unmethylated DNA sequences, which was related to tumour heterogeneity or to the occurrence of intermingled non-neoplastic cells. The epigenetic profile of *RASSF1A*, *E-Cadherin* and *p16INK4A* in CRC is shown in Table 2b.

#### *RASSF1A*

Out of 202 tumours, *RASSF1A* was methylated in 40 (20%) tumours, the majority occurring in M+ tumours (*P*=0.021). No other association was found between *RASSF1A* methylation and the clinico-pathological parameters under study.

#### *E-Cadherin*

Out of 202 cases, *E-Cadherin* methylation was detected in 88 (44%) cases. *E-Cadherin* methylation was more frequently detectable in the right colon (*P*=0.011) and in older patients (*P*=0.002). *E-Cadherin* methylation was also associated with *RASSF1A* methylation (*P*=0.047). All these variables retained a statistical significance in multivariate analysis (right side: OR 0.59, 95% CI, 0.40–0.87, *P*=0.007; older age: OR 1.039, 95% CI, 1.01–1.07, *P*=0.004; *RASSF1A* methylation: OR 2.11, 95% CI, 1.01–4.41, *P*=0.047).

#### *p16INK4A*

Out of 202 cases, *p16INK4A* promoter methylation was found in 67 (33%) cases. *p16INK4A* methylation was significantly associated with M+ tumours (*P*<0.001). *p16INK4A* methylation was associated with *B-Raf* mutations (*P*<0.001), *RASSF1A* (*P*=0.004) and *E-Cadherin* (*P*=0.040) methylation. *p16INK4A* methylation was more frequently detectable in *p53* mutated cases (*P*=0.075). Multivariate analysis showed that gene methylation was statistically associated with M+ tumours (OR 3.48, 95% CI, 1.66–7.26, *P*=0.001) and with *RASSF1A* methylation (OR 2.29, 95% CI, 1.07–4.89, *P*=0.032).

### Coincidental genetic and epigenetic changes

Out of 202 CRCs, thirty-four (17%) were free from molecular abnormalities and with no association with M+ *vs* M− tumours. Regardless the type of molecular abnormalities, 68 cases (34%) showed one molecular alteration, 57 cases (28%) two molecular alterations and 43 cases (21%) ⩾3 molecular alterations. No statistical association was seen between M+ and M− CRCs showing one or two gene alterations. Conversely, the frequency of CRCs in the group showing three or more alterations was higher in M+ than in M− tumours (34/130=26% *vs* 9/72=12%; *P*=0.023). In the latter (⩾3 abnormalities) group, the most frequent combination of genes was one genetic and two epigenetic alterations, particularly *K-Ras* mutations (18/34=53%), with gene methylation equally affecting the three genes under study (*RASSF1A*, *E-Cadherin* and *p16INK4A*). Coincidental methylation of these genes was also documented in 12 cases, 11 of whose were M+ CRCs (*P*=0.042).

### Multivariate analysis of variables associated with M+ tumours

Among the variables associated with M+ cancers in univariate analysis (*K-Ras* mutation, *p16INK4A* methylation, *RASSF1A* methylation, younger age and grading), multivariate analysis showed that only *K-Ras* mutations (OR 2.98, 95% CI, 1.37–6.46, *P*=0.006), *p16INK4A* methylation (OR 4.58, 95% CI, 2.14–9.76, *P*<0.001) and younger age (OR 0.95, 95% CI, 0.92–0.98, *P*<0.001) retained a statistically significant value.

## DISCUSSION

Aim of this study was to characterise genetic and epigenetic molecular alterations occurring in primary advanced M+ and M− CRCs.

The model of CRC relies upon the sequential accumulation of genetic and epigenetic changes but, in our study, 17% of cases had no abnormalities in the investigated genes (*K-Ras*, *B-Raf*, *p53*, *RASSF1A*, *E-Cadherin*, *p16INK4A*). These data are in agreement with previous studies examining different sets of genes. Smith *et al* (2002) investigated *K-Ras*, *p53* and APC in 106 cases and 10% CRC were reported free of molecular abnormalities. Lee *et al* (2004) examined promoter methylation of 12 different genes in 149 CRCs and found that 9% were unmethylated. Chiang *et al* (2004) also demonstrated that the three key mutations (APC, *K-Ras* and *p53*) are rarely coincidental in 122 sporadic CRCs. All these findings suggest that alterations in the genes mostly involved in colorectal carcinogenesis are not always requested for tumour progression.

We found 27% *K-Ras* mutations in the whole series and a statistical association with M+ tumours (univariate analysis *P*=0.016; multivariate analysis *P*=0.019), suggesting *K-Ras* activation as an important determinant of extracolic tumour spread. Gonzalez-Aguilera *et al* (2004) found increased *K-Ras* mutations in tumours with advanced Duke's stage (A 22%, B 27%, C 38% and D 33%), particularly when grouping M+ (46%) *vs* M− tumours (26%). Aberrant activation of *K-Ras* has been implicated in facilitating all aspects of a malignant phenotype, including proliferation, invasion and metastases by Ras effectors such as PIK3, Raf/MEK/ERK cascade and Rho GTPases. Recently, some authors proposed that epithelial cells with activated Ras signalling require the cooperation of other signalling pathway to became metastogenic (Grunert *et al*, 2003) and the oncogenic Ras is thought to cooperates with several other proto-oncogenes such as *p53* and *p16INK4A* (Serrano *et al*, 1996; Aguirre *et al*, 2003). This cooperation between *Ras* and other genes is likely to occur in CRC progression because, in our study, only 6% of tumours showed the exclusive presence of *K-Ras* mutation. *E-Cadherin* promoter methylation was prevalent in the right colon (univariate analysis *P*=0.011; multivariate analysis *P*=0.007), so that coincidental *K-Ras* mutations and *E-Cadherin* methylation frequently occurred in proximal (27%) rather than left colon (5%) and rectum (8%) (*P*<0.001). Whether *E-Cadherin* methylation and *K-Ras* mutation cooperate in right side colonic carcinogenesis and progression have to be confirmed in larger series. *B-Raf* was rarely found mutated in our cases and never in conjunction with *K-Ras*. As previously noted (Rajagopalan *et al*, 2002; Yuen *et al*, 2002), *K-Ras* and *B-Raf* mutations appear to be mutually exclusive, confirming previous suggestions that they have similar biological targets in the RAS-RAF-MEK-ERK-MAP kinase signalling pathway (Yuen *et al*, 2002). A small proportion of cases (10/202=5%) showed both *K-Ras* mutations and *RASSF1A* methylation; interestingly all these cases were M+ (*P*=0.016). Whether alterations in both these genes may be able to confer a more aggressive phenotype remains to be established in larger series.

In our study, *p53* abnormalities were not prevalent in M+ tumours suggesting a limited role of *p53* in CRC metastatisation. Different results were reported by Goh *et al* (1999) that used only SSCP to detect *p53* mutations.

Among tumour suppressor genes showing DNA methylation, *p16INK4A* seems to play a major role in the metastogenic phenotype of primary CRCs. Methylation was seen in 33% of the whole series and it was, like *K-Ras* mutation, significantly associated with a M+ phenotype (univariate analysis *P*=0.011; multivariate analysis *P*=0.001). A strong correlation between staging and *p16INK4A* methylation was found also by Yi *et al* (2001); loss of *p16INK4A* expression has been previously associated with lymph-node metastases (Kim *et al*, 2005a) and more advanced stage (Jeong *et al*, 2005). Altogether, these results suggest that *p16INK4A* methylation might link to a more malignant phenotype in CRC; interestingly Liang *et al* (1999) recently reported an association between *p16INK4A* methylation and shorter survival of CRC. Coincidental alterations of *K-Ras* and *p16INK4A* were mainly found in M+ tumours (2/67 M− tumours *vs* 17/139 M+ tumours; *P*=0.034), suggesting that abnormalities in these pathways could cooperate for a more aggressive phenotype, as previously supported by Serrano *et al* (1997).

Another focus of this study was to examine the interactions between genetic and epigenetic alterations in the induction of a metastogenic phenotype. Regardless the type of molecular abnormalities, tumours with three or more but not those with less than three, were significantly more prevalent in the group of M+ tumours (*P*=0.023). This means that the genes under study can confer a more aggressive phenotype when molecular changes deregulate at least three of them. This hypothesis is in keeping with a stepwise model of progression of CRC. Given that a fraction of M− CRCs (13%) already harbours three or more molecular changes, we are currently analysing this cohort to ascertain whether these cases will develop metachronous liver metastasis at an increase rate as compared to M− CRCs with <3 molecular changes. The group of M+ tumours showing three or more abnormal genes was characterised by a single genetic mutation (usually *K-Ras*; 53%) and by two epigenetic alterations (equally affecting *p16INK4A*, *RASSF1A* and *E-Cadherin*). Notably, Toyota *et al* (2000) found *K-Ras* mutations significantly prevalent in CRCs with the so called methylator phenotype (CIMP) compared with CIMP negative cases. A stepwise increase in the number of methylated genes was observed with lesion progression through the stages of multistep colorectal carcinogenesis by Lee *et al* (2004). Our study showed that coincidental methylation of the three genes (*RASSF1A*, *E-Cadherin* and *p16INK4A*) was restricted to M+ tumours (*P*=0.042). It is unclear why advanced and M+ CRCs showed a high degree of methylation: perhaps advanced tumours inherited a major biological aggressiveness or were simply more likely to become methylated. Notably, patients showing three methylated genes were also of older age (73.83 *vs* 65.60 years; *P*=0.017), in keeping with the suggestion put forwards by Toyota and Issa (1999) that age-related methylation might contribute to the sharp increase in cancer risk after the age of 60 years.

Interestingly, in the present study, younger age was statistically associated to M+ tumours. This is likely due to internal selection for surgical treatment, being the older age a limiting factor for radical surgical resection (Leporrier *et al*, 2006).

In conclusion, analysis of a panel of genes provided to detect some genetic and epigenetic molecular differences between M+ and M− primary CRCs. A set of genetic and epigenetic molecular abnormalities is a feature of primary M+ CRCs likely conferring the ability for tumour extracolic spread and determining markers for aggressive behaviour and potential therapeutic targets.

## Figures and Tables

**Table 1:** Clinico-pathological features of the series under study as related to M+ and M− tumours

	**All patients**	**M−**	**M+**	
	***n*=202 (100%)**	***n*=72 (100%)**	***n*=130 (100%)**	**Univariate analysis**
Age (years±s.d.)	66.05±11.66	69.40±11.44	64.2±11.40	*P*=0.002
				
*Sex*				
F	81 (40%)	26 (36%)	55 (42%)	NS
M	121 (60%)	46 (64%)	75 (58%)	
				
*Location*				
R	71 (35%)	23 (32%)	48 (37%)	NS
L	78 (39%)	30 (42%)	48 (37%)	
Re	53 (26%)	19 (26%)	34 (26%)	
				
*Grading*				
G1	14 (7%)	9 (13%)	5 (4%)	*P*=0.069
G2	145 (72%)	47 (65%)	98 (75%)	
G3	43 (21%)	16 (22%)	27 (21%)	

F=female; L=left side; M=male; M+=metastatic tumours; M−=nonmetastatic tumours; NS=not significant; R=right side; Re=rectum; s.d.=standard deviation.

**Table 2a:** Genetic alterations: mutation frequencies for *K-Ras*, *B-Raf* and *p53*

	**All cases**	***K-Ras***		***B-Raf***		***p53***	
	**No.**	**No. (%)**		**No. (%)**		**No. (%)**	
All patients	202	54 (27%)		7 (3%)		65 (32%)	
Age (years±s.d.)	66.05±11.66	67.16±11.56	NS	64.42±13.50	NS	65.23±12.17	NS
*Sex*							
F	81	24 (30%)	NS	2 (2%)	NS	22 (27%)	NS
M	121	30 (25%)		5 (4%)		43 (35%)	
							
*Tumour*							
M−	72	12 (17%)	*P*=0.016	1 (1%)	NS	26 (36%)	NS
M+	130	42 (32%)		6 (5%)		39 (23%)	
							
*Location*							
R	71	25 (35%)		5 (7%)		23 (32%)	
L	78	15 (19%)	*P*=0.088	1 (1%)	NS	23 (29%)	NS
Re	53	14 (26%)		1 (2%)		19 (36%)	
							
*Grading*							
G1	14	5 (36%)		—		4 (29%)	
G2	145	38 (26%)	NS	4 (3%)	NS	48 (33%)	NS
G3	43	11 (26%)		3 (7%)		13 (30%)	

F=female; L=left side; M=male; M+=metastatic tumours; M−=nonmetastatic tumours; NS=not significant; R=right side; Re=rectum; s.d.=standard deviation.

**Table 2b:** Epigenetic alterations: promoter methylation frequencies for *RASSF1A*, *E-Cadherin* and *p16INK4A*

	**All cases**	***RASSF1A***		**E-CAD**		***p16INK4A***	
	**No.**	**No. (%)**		**No. (%)**		**No. (%)**	
All patients	202	40 (20%)		88 (44%)		67 (33%)	
Age (years±s.d.)	66.05±11.66	66.75±12.52	NS	68.86±10.63^a^	NS	67.25±12.02	NS
*Sex*							
F	81	18 (22%)	NS	34 (42%)	NS	27 (33%)	NS
M	121	22 (18%)		54 (45%)		40 (33%)	
							
*Tumour*							
M−	72	8 (11%)	*P*=0.021	34 (47%)	NS	12 (17%)	*P*<0.001
M+	130	32 (25%)		54 (41%)		55(42%)	
							
*Location*							
R	71	14 (20%)		41 (58%)		24 (34%)	
L	78	15 (19%)	NS	29 (37%)	*P*=0.011	27 (35%)	NS
Re	53	11 (21%)		18 (34%)		16 (30%)	
							
*Grading*							
G1	14	3 (21%)		7 (50%)		5 (36%)	
G2	145	28 (19%)	NS	65 (45%)	NS	48 (33%)	NS
G3	43	9 (21%)		16 (37%)		14 (33%)	

F=female; L=left side; M=male; M+=metastatic tumours; M−=nonmetastatic tumours; NS=not significant; R=right side; Re=rectum; s.d.=standard deviation.

a Methylated *vs* nonmethylated=68.86±10.63 *vs* 63.89±11.99 (*P*=0.002).
