# Supplementary figures and images for: Genetic and epigenetic changes in primary metastatic and nonmetastatic colorectal cancer
Source: Br J Cancer. 2006 Sep 12;95(8):1101–7. doi: 10.1038/sj.bjc.6603337 (PMC2360724; doi:10.1038/sj.bjc.6603337)

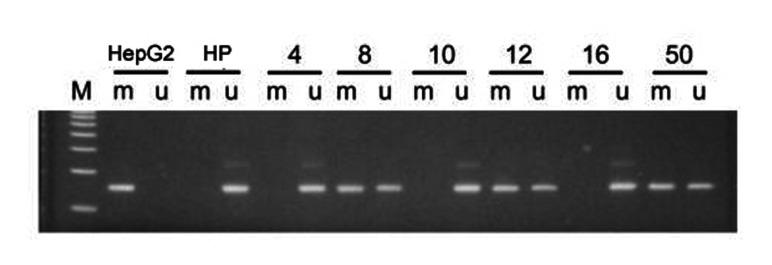

Supplement: Supplementary Figure 1 [file 95-6603337x1.jpg]
