# Supplementary material for: Genetic and epigenetic changes in primary metastatic and nonmetastatic colorectal cancer
Source: Br J Cancer. 2006 Sep 12;95(8):1101–7. doi: 10.1038/sj.bjc.6603337 (PMC2360724; doi:10.1038/sj.bjc.6603337)
Supplement: Supplementary Table 1 [file 95-6603337x2.doc]

| **TABLE 1 (Supplementary Appendix) Primers sequence and PCR conditions for *p53*, *RASSF1A*, *E-Cadherin* and *p16INK4A*** | | | | | |
| --- | --- | --- | --- | --- | --- |
| **Primers** | **sequence** | **denaturation** | **annealing** | **elongation** | **n° cycles** |
| *p53* ex 5 sense | ACTTTCAACTCTGTCTCCTTCCTCTTC | 94°C 20 sec | 56°C 20 sec | 72°C 40 sec | 40 |
| *p53* ex 5 antisense | CAGCCCTGTCGTCTCTCCAG |
| *p53* ex 6 sense | CCAGAGACCCCAGTTGCAAA | 94°C 20 sec | 55°C 20 sec | 72°C 40 sec | 40 |
| *p53* ex 6 antisense | CCAGAGACCCCAGTTGCAAA |
| *p53* ex 7 sense | TCATCTTGGGCCTGTGTTATCTC | 94°C 20 sec | 58°C 20 sec | 72°C 40 sec | 40 |
| *p53* ex 7 antisense | GTGCAGGGTGGCAAGTGG |
| *p53* ex 8 sense | CCTCTTGCTTCTCTTTTCCTATTCCT | 94°C 20 sec | 53°C 20 sec | 72°C 40 sec | 40 |
| *p53* ex 8 antisense | CGCTTCTTGTCCTGCTTGCT |
| *RASSF1A* MSP sense | GGGTTTTATAGTTTTTGTATTTAGGTT | 94°C 15 sec | 48°C 15 sec | 72°C 30 sec | 20 |
| *RASSF1A* MSP antisense | AACTCAATAAACTCAAACTCCCC |
| *RASSF1A* U. sense | TGTGTGGTTTTTTTTAGTTTTTTTTTGTTG | 94°C 15 sec | 55°C 15 sec | 72°C 30 sec | 25 |
| *RASSF1A* U. antisense | CCCAACATAACCCAATTAAACCA |
| *RASSF1A* M. sense | CGGTTTTTTTTAGTTTTTTTTCGTCG |
| *RASSF1A* M. antisense | TAACTTTAAAGGCTAACAAACGCGAA |
| *E-Cadherin* MSP sense | GTTGTGGTYGGTAGGTGAATT | 94°C 15 sec | 48°C 15 sec | 72°C 30 sec | 20 |
| *E-Cadherin* MSP antisense | ACTCCAAAAACCCATAACTAACC |
| *E-Cadherin* U. sense | GTTGTAGTTATGTATTTTTTTTTAGTGGTGTTGG | 94°C 15 sec | 56°C 15 sec | 72°C 30 sec | 25 |
| *E-Cadherin* U. antisense | ACCAAAAACACCAAACAAAAACAAACA |
| *E-Cadherin* M. sense | TACGTATTTTTTTTTAGTGGCGTCGG |
| *E-Cadherin* M. antisense | AACGCCGAACGAAAACAAACG |
| *p16INK4A* MSP sense | CAGGGGTTGGTTGGTTATTAG | 94°C 30 sec | 52°C 30 sec | 72°C 30 sec | 20 |
| *p16INK4A* MSP antisense | AAACCCTCTACCCACCTAAATC |
| *p16INK4A* U. sense | TTATTAGAGGGTGGGGTGGATTGT | 94°C 30 sec | 59°C 30 sec | 72°C 30 sec | 25 |
| *p16INK4A* U. antisense | CAACCCCAAACCACAACCATAA |
| *p16INK4A* M. sense | TTATTAGAGGGTGGGGCGGATCGC | 94°C 30 sec | 65°C 30 sec | 72°C 30 sec | 25 |
| *p16INK4A* M. antisense | GACCCCGAACCGCGACCGTAA |
| Abbreviations: MSP= Methylation Specific PCR primers, U= unmethylated DNA specific primers, M= methylated DNA specific primers | | | | | |
